# Physical Incompatibility between Vancomycin and Viscoelastic Mimicking Acute Endophthalmitis: The First Report

**DOI:** 10.1155/2019/6341694

**Published:** 2019-02-04

**Authors:** Biljana Kuzmanović Elabjer, Dean Šarić, Mladen Bušić, Mirjana Bjeloš, Andrej Pleše

**Affiliations:** University Eye Clinic, Faculty of Dental Medicine and Health Care Osijek, Faculty of Medicine Osijek, University Josip Juraj Strossmayer in Osijek, University Hospital “Sveti Duh”, Zagreb, Croatia

## Abstract

This is the first documentation of vancomycin precipitation with viscoelastic in the anterior chamber of the eye. A 34-year-old white male underwent uneventful penetrating keratoplasty. Intracameral instillation of 1 mg/0.1 mL of vancomycin followed no attempts of meticulous viscoelastic irrigation. Six hours later thick white material in the anterior chamber was sedimented. The following criteria excluded the diagnosis of endophthalmitis and TASS: clear and transparent anterior chamber and vitreous body, the absence of ciliary injection and corneal oedema, and unremarkable laboratory tests' results. This iatrogenic complication mimicking endophthalmitis does not require any specific management and should be acknowledged in guidelines for prevention and treatment of endophthalmitis. The objective of this paper is to alert colleagues to this iatrogenic complication of vancomycin mimicking endophthalmitis. Whether this condition should be labelled as positive or negative demands further investigation. As vancomycin is a time-dependent antibiotic, it is possible that this precipitate could serve as a slowly releasing drug depot and viscoelastic as a vehicle for precipitation. This being the case, investigation is needed to analyse its potential to precipitate with another dispersive and cohesive viscoelasticity.

## 1. Introduction

Vancomycin hydrochloride is a glycopeptide antibiotic used in ophthalmic surgery for postoperative endophthalmitis prophylaxis [1]. It precipitates with gelatine fluid, ciprofloxacin, cloxacillin, and ceftazidime [2–5]. According to medical literature this is the first publication of precipitation of vancomycin with residual viscoelastic (hydroxypropyl methylcellulose IP 2% w/v, Aurovisc®, Madurai, India) in anterior chamber.

## 2. Case Report

**Figure 1:**
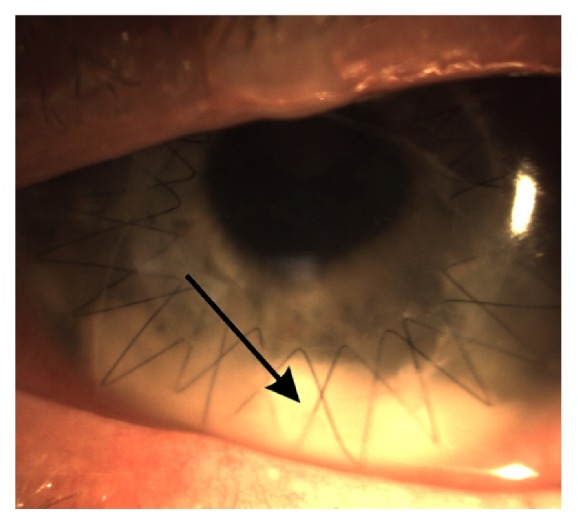
The right eye of 34-year-old white male on day zero of the penetrating keratoplasty. Note absence of the corneal oedema. White material at the bottom (black arrow), irregularly bordered superiorly to the rest of the clear anterior chamber.

A 34-year-old white healthy male underwent uneventful penetrating keratoplasty for keratoconus of ABCD stage 4 [6]. Preoperatively antisepsis of the periocular skin and eye with 10% povidone-iodine solution was applied for three minutes. The graft was sutured with 10-0 double running nylon (ETHILON® Nylon Suture, Ethicon, USA). For the prophylaxis of endophthalmitis, in this patient vancomycin was indicated due to the allergy to beta-lactam antibiotics and 1 mg/0.1 mL was instilled intracamerally. The vancomycin powder (Vancomycin Kabi 500 mg, Xellia Pharmaceuticals ApS, Denmark) was reconstituted and diluted with 0.9% sodium chloride injection. Six hours after the surgery the patient complained of pain in the operated eye. Photophobia, blepharospasm, and pale lid oedema were present with pinhole visual acuity of logMAR 1.0. No ciliary injection or corneal oedema was present. Graft was secured to the host cornea with equal tension along the suture and negative Seidel test. White, cheese-like material, sedimented and irregularly bordered superiorly to the rest of the clear anterior chamber, was found [Figure 1].

Tyndall was negative, pupil round, and reactive. The lens and the vitreous body were clear. Applanation tonometry was 11 mmHg. Ultrasound documented no pathology of the eye and orbit. The pain decreased on tetracaine drops (Tetrakain® 0.5%, Gradska ljekarna, Zagreb, Croatia). Laboratory tests' results (complete blood count with differential and erythrocyte sedimentation rate) were unremarkable. Under the strong presumption that vancomycin precipitated in the residual viscoelastic no attempts to lavage the anterior chamber were undertaken. Topical dexamethasone with antibiotic (Maxitrol, Alcon Cusi SA, El Masnou, Barcelona, Spain; SA, Alcon-Couvreur NV, Puurs, Belgium) was administered q.i.d. Ophthalmological follow-up was performed hourly for seven hours until the pain resolved. Complete dissolution of precipitates ensued 19 hours following the surgery.

The case report was written with the approval of the institutional research ethics committee, complying with the tenets of the Declaration of Helsinki.

## 3. Discussion

Patient complaining of pain in the operated eye on the day of surgery with thick white precipitate sedimented in the anterior chamber is an alert for the ophthalmologist to be on standby. Apart from the most obvious infectious endophthalmitis, foreign particles used in surgery and toxic anterior segment syndrome (TASS) must be considered in differential diagnosis [7, 8]. The main distinctions between infectious endophthalmitis and TASS are onset and progression of symptoms as well as the presence of pain and vitritis [7]. However, in both conditions corneal involvement and major inflammatory reaction in the anterior chamber are present [7]. The following criteria excluded the diagnoses of endophthalmitis and TASS: clear and transparent anterior chamber and vitreous body, absence of ciliary injection and corneal oedema, and unremarkable laboratory tests' results as well as the fact that the pain decreased on the tetracaine drops. The material in the anterior chamber, if not of inflammatory origin, had to be iatrogenic. Namely, no meticulous aspiration of viscoelastic was performed due to intraoperative tendency of anterior chamber shallowing. In Aurovisc® instructions for use leaflet acknowledged that the concurrent presence of medication in the chamber or associated ocular structures may interact with Aurovisc® to cause clouding.

It is documented that vancomycin has precipitative properties with different substances, especially those in gelatinous form [2]. Aurovisc® is hydroxyl propyl methyl cellulose viscoelastic solution of 3000–4500 cps at 27°C containing no sodium carbonate. It was advocated that vancomycin and ceftazidime precipitate due to the presence of sodium carbonate in ceftazidime [9]. When vancomycin and ceftazidime were mixed, precipitates formed at a concentration of 10 mg/mL for vancomycin, the same as in this report. However, the precipitation was documented regardless of sodium carbonate presence, favouring alkaline pH as the major element in the precipitation process [5, 10]. The vancomycin–ceftazidime precipitate manifested pH of 6.2, while vancomycin itself, as a salt of hydrochloric acid, is set to pH range 2.5–4.5 [2, 10]. In addition, the study that is more recent evidenced no precipitation of the two drugs, when the drugs were administered in the media one after the complete diffusion of the other [5].

**Figure 2:**
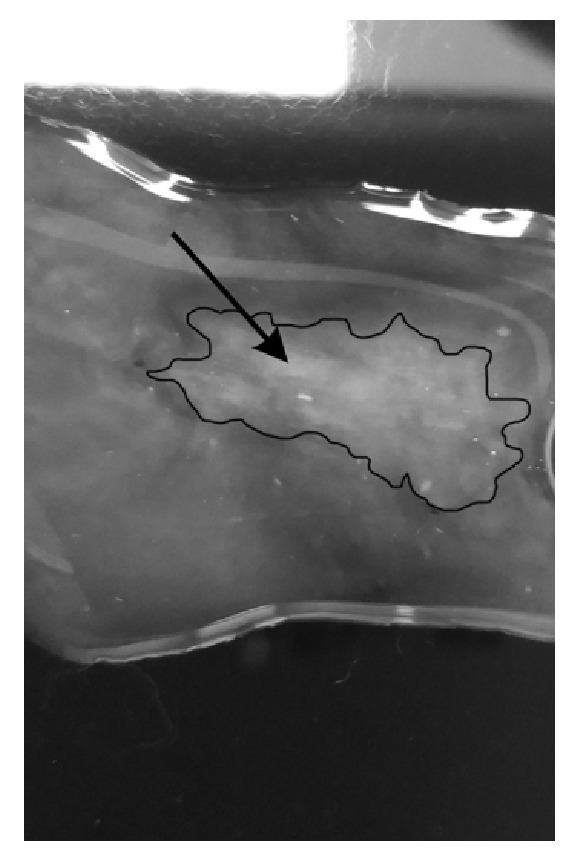
In vitro on the glass slide clear vancomycin at the concentration of 1 mg/0.1 mL was instilled in a clear Aurovisc® (black outline). Note opacification (black arrow).

Experimentally, instant precipitation occurred [Figure 2], in vitro on the glass slide when a drop of 1 mg / 0.1 mL vancomycin was added in a clear Aurovisc®.

In our patient, precipitates sedimented in anterior chamber dissolved spontaneously, like the precipitates in vitreous cavity reported earlier [11]. The time required, due to different hemodynamics between vitreous cavity and anterior chamber, was markedly shorter.

In conclusion, this is the first documentation of vancomycin precipitation with viscoelastic in the anterior chamber of the eye. This should be acknowledged in guidelines for prevention and treatment of endophthalmitis as this iatrogenic complication mimicking endophthalmitis does not require any specific management.

The incompatibility developed when the vancomycin drug was administered at the recommended concentration for endophthalmitis prophylaxis, advocating pH and dispersion of vancomycin as two major factors in precipitation process. The objective of this paper is to alert colleagues to this iatrogenic complication of vancomycin mimicking endophthalmitis. Whether this complication should be labelled as positive or negative demands further investigation. As vancomycin is a time-dependent antibiotic, failing to achieve an efficient bactericidal level over at least 11 h, it is possible that this precipitate could serve as a slowly releasing drug depot and viscoelastic as a vehicle for precipitation [10, 12, 13]. This being the case, research is needed to analyse its potential to precipitate with other dispersive and cohesive viscoelastic.

## Additional Points

*Key Message*. This is the first documentation of vancomycin precipitation with viscoelastic in the anterior chamber of the eye. This should be acknowledged in guidelines for prevention and treatment of endophthalmitis as this iatrogenic complication mimicking endophthalmitis does not require any specific management.
